# Reflections on pneumonia in the tropics

**DOI:** 10.15172/pneu.2014.4/416

**Published:** 2014-12-01

**Authors:** Michael P. Alpers

**Affiliations:** Faculty of Health Sciences, Curtn University, Perth, Australia Room 108, Health Research Campus, Shenton Park, GPO Box U1987,

**Keywords:** pneumonia, nasopharyngeal carriage, pneumococcal polysaccharide vaccine, ecosystem of infection, community engagement

## Abstract

This review of pneumonia in the tropics is based on experience with respiratory infectons in Papua New Guinea since the 1970s. It discusses ideas, principles, historical aspects of pneumonia research and the need to work with people in the community. In order to understand pneumonia in a tropical setng and evaluate new interventons it is essental to study the ecosystem of the causatve infectons, within the host and the community and between interactng microorganisms. Vaccines are much-needed preventve tools, and for pneumonia in a highly endemic setng the preventon of severe and fatal disease takes priority over the preventon of infecton. In this setng mild infecton plays an important role in preventng severe disease. For achieving long-term sustainable outcomes, sometmes ‘less is more’. A multpronged approach is required to control and prevent pneumonia, and in devising new ways of doing so. This includes appropriate and accessible clinical care, a clean, smoke-free environment, good nutriton and a range of vaccines. Also required are persistent advocacy from the global scientfc community and strong engagement with and by the communites that bear the burden of disease. Beter health care must be pursued in conjuncton with raising literacy rates and reducing poverty.

The following observations and reflections derive from my experiences working with many creative and committed scientists across a range of disciplines investigating pneumonia and other tropical infectious diseases at the Papua New Guinea Institute of Medical Research, of which I was the Director from 1977 to 2000.

One of the first things we learned in our studies of infectious disease in Papua New Guinea was that to understand an infection we had to investigate the ecosystem in which it occurred. Later we learned that the results of an intervention depended on the specifics of the ecosystem in which it was applied.

Following the pioneering work of Robert Douglas [1] and Ian Riley [2] in the 1970s we undertook studies on pneumonia [3] within a comprehensive set of research programs [4]. We looked for the invading organisms in the blood and lungs [5, 6]. We also investigated the ecosystem inhabited by these organisms, which at the time was considered an unusual approach. We studied the nasopharynx [7, 8], the first acquisition of pathogenic organisms after birth [9] and the complex relationships of acquisition, carriage and invasiveness [10]. Bacterial pathogens were acquired remarkably early in life and high levels of bacterial carriage persisted in the nasopharynx throughout childhood. We also studied the ecosystem of the household and the response to an intervention against the principal source of respiratory pathogens in the household, namely, chronic bronchitis in adults [11].

*Streptococcus pneumoniae* (pneumococcus) and *Haemophilus influenzae*, together with a number of viruses such as respiratory syncytial virus, were the principal respiratory pathogens found. The pneumococcus and *H. influenzae* were also important causes of meningitis [12–14]. These organisms were of established significance world-wide and the subject of much past and ongoing research. The first trial of a 7-valent pneumococcal conjugate vaccine in the United States demonstrated a powerful specific effect in children [15], which was widely acclaimed, and a dramatic effect of herd immunity in protecting unvaccinated adults from pneumococcal disease [16]. These encouraging results were demonstrated in the population for which the vaccine was designed. In other populations, however, the outcome was somewhat different [17]. Such a difference in another cultural, ethnic and ecological setting was entirely to be expected, at least by those who worked in tropical communities of the Third World.

The conjugate pneumococcal vaccine has not been rolled out yet in Papua New Guinea though a country-wide program will be established with the 13-valent vaccine in 2014. We know that the coverage of pathogenic serotypes by the vaccine will be less than in the First World. We can expect significant replacement infection and disease, probably more than occurred in Alaskan Native Americans [17]. We do not expect to get herd immunity, since this can only occur where the community source of infection is the group that has been immunised, in this case young children. Where, in contrast, the route of transmission goes the other way, and children are principally infected by adults living in their household, adults will not be protected by immunising infants. Indeed, any gaps in the nasopharyngeal niche of children created by vaccination will readily be filled by locally circulating pathogenic types not in the vaccine. That is why, apart from its benefit in improving the health of adults, we were keen to implement the oral vaccine — or immune modulator — that reduced bronchial bacterial load [11], and the health services of Papua New Guinea were strongly supportive of this initiative. Unfortunately the manufacture of the vaccine ceased. Further studies of a new formulation have since been undertaken and we still hope that it may become available again [18].

In trying to modify the ecosystem to the benefit of the host it may be more sustainable in the long run to use a minor perturbation rather than a major disruption that evokes a powerful reactive adjustment to maintain ecological stability. In this respect the use of a vaccine that does not affect nasopharyngeal carriage and provides immunity against a broad range of pathogenic serotypes may be ultimately advantageous. On first principles there is an advantage in filling the niche of infection in the nasopharynx with organisms to which the host is immune, to the extent of being able to mount a rapid and effective response against invasive disease. These bacteria will reduce nasopharyngeal infection by other competing organisms and if they invade will be dealt with by host immunity before they cause severe and fatal disease. The polysaccharide pneumococcal vaccine has been shown to provide such protection [19]. This benefit can be achieved for children aged 9 months and over — the belief that the polysaccharide vaccine is non-immunogenic and ineffective in children under the age of 2 years is still trotted out as dogma, even though in tropical populations of the Third World it is manifestly untrue [20, 21]. For children under the age of 9 months some form of protein-based vaccine is required, whether a set of protein-polysaccharide conjugates covering specific serotypes, a combination of species-specific pneumococcal proteins or a whole cell vaccine. We have to expect that counterstrategies by the pneumococcus, either replacement with other serotypes or proliferation of variant strains, will diminish the initial vaccine efficacy. Some bacterial vaccine interventions have been singularly successful and we must keep trying with established and new technologies, such as reverse vaccinology, but we also need to temper our optimism with humility. In our human arrogance we tend to think of bacteria as primitive organisms and forget that they have been evolving and adapting in a very competitive world for aeons longer than we have.

For *H. influenzae*, type b stands out as the most virulent serotype and *H. influenzae* type b (Hib) vaccine has had dramatic effects in reducing Hib disease wherever it has been used, including Papua New Guinea. However, Hib is not the only serotype of *H. influenzae* causing disease [22] and type a disease may be becoming more common in Papua New Guinea. Whether this proves to be a serious problem will be determined by ongoing surveillance at the Papua New Guinea Institute of Medical Research. Nontypeable *H. influenzae* are also important organisms whose role in disease requires further investigation [23]. We do not understand the complexities of infection and bacterial association among different species and strains, pathogenic and non-pathogenic, within the nasopharynx but we do know that if a niche is empty it will not remain so but be filled by other organisms.

Another important principle that we learned in Papua New Guinea is the importance of mild infection [24]. This was the principle of variolation, which predated the concept of attenuated infection through vaccination, which is a cognate principle and generally a safer practice. However, in the absence of a vaccine, mild infection plays an important role in preventing fatal disease. It cannot be guaranteed but can be promoted by strategies to reduce the infecting dose and strengthen the immune system, through various environmental and nutritional improvements. Chronic mild infection is particularly important in malaria, where it helps to maintain adult immunity in malaria-endemic areas. The combination blood-stage vaccine against malaria that we devised and tested was intended not to have any effect on infection but to prevent severe disease and death from malaria. This principle was designed for the vaccination of children in an endemic area and proof of the principle was demonstrated in a Phase 2b vaccine trial where density of infection was used as a surrogate for disease severity [25, 26]. The same principle applied to our work on pneumonia. The trial of the 23-valent pneumococcal polysaccharide vaccine showed no effect on mild pneumonia but a 58% efficacy against death from pneumonia in children aged 6 months to 5 years and, because of the importance of pneumonia as a cause of death in this population, a 30% reduction in all-cause mortality [19]. An effect on severe morbidity was also demonstrated [27] but only when the burden was high during a pneumonia epidemic. Even in children aged 6 months to 2 years there were significant effects of the vaccine.

Because of the high burden of severe disease and mortality from pneumonia in young children we wanted to start administering the vaccine in Papua New Guinea as soon as possible, but the country could not afford it. However, its cost of manufacture was not high and, given a global market, we expected that the price could have been brought down to an affordable level. Thus our first move after conducting this trial in the Papua New Guinean highlands was to try and have it replicated elsewhere. This was done with the full support of the World Health Organization (WHO), which had supported the trial itself. Somewhat ironically, because of a high expectation of its efficacy, WHO had imposed methodological constraints on the study, insisting on sequential analysis so that at the first evidence of efficacy the trial could be stopped and the vaccine given to the control group. This gave a clear outcome but significantly compromised our ability to undertake more in-depth analysis of the results. With WHO support a replicate trial was designed in The Gambia. However, our colleagues engaged in the development of a conjugate pneumococcal vaccine persuaded the funders of this trial that it was not worth pursuing because the more powerful conjugate pneumococcal vaccine was ‘just around the corner’. This was in 1989: 24 years later Papua New Guinean children still have not received the benefit of pneumococcal vaccination. During that period how many lives could have been saved from the use of the 23-valent pneumococcal polysaccharide vaccine in Papua New Guinea and in other countries of the Third World with similar ecological characteristics?

The development of vaccines against complex infections has much in common with the deployment of antibiotics. The original hype that the use of antibiotics would soon eliminate infectious disease, an opinion expressed even by leaders in biological science, was based on ignorance of the adaptability of microorganisms. Even scientists seem to forget that bacteria have a long experience of dealing with antibiotics in their environment. In the 1970s we conducted a study in a remote part of Papua New Guinea looking for bacterial resistance to antibiotics that had never been used in the participating communities. As a result we isolated several gut organisms that were resistant to these antibiotics. Though this was what some of our team had predicted, the finding was at the time considered controversial, to the unfortunate extent that our more conservative colleagues would not agree to its publication.

In some infections, such as with a specific virus that infects only humans, eradication is theoretically and, indeed, in the case of smallpox, practically possible. This is not so for complex infections and diseases such as malaria or pneumonia. If the infection has low endemicity or is epidemic it may be possible to eliminate it in that location. Where eradication is not possible or has not been achieved, elimination is dangerous, since it sets up an open niche with absent or waning host immunity, or creates other kinds of vulnerability. This is true across a range of infections from polio to malaria. Elimination is worth it if constant and rigorous surveillance, which does not come cheaply, can be provided to prevent the reintroduction of the infection and deal immediately with any resurgence of disease. Where overall elimination is not possible, as with respiratory infections, and good control is the ideal, it may be a sound investment to include in the long-term strategy a component that leads to minimal perturbation of the relevant ecosystem. In this respect we may anticipate a continuing future role for polysaccharide pneumococcal vaccination in the control of pneumonia. This is especially so if the vaccine is offered at a price that low-income countries can sustainably afford — which is, unfortunately, unlikely to happen with conjugate vaccines. The multiplicity of pathogenic serotypes in the ecosystem in tropical communities is a significant risk factor and warrants pneumococcal capsular vaccines of high valency; the 23-valent vaccine could possibly be expanded for this purpose and the dose of each component reduced. However, this need may change when the long-awaited species-specific protein vaccines have been licensed and their impacts evaluated: dare we hope that this is ‘just around the corner’?

In recent studies in Papua New Guinea the 23-valent pneumococcal polysaccharide vaccine has been used to provide boosting (at age 9 months) of responses initiated at birth or in early infancy by conjugate vaccine [28]. The results have shown that neonatal immunisation is safe and immunogenic [29] and immunologically safe, with good T-cell responses induced [30]. Immunisation at birth has two main advantages: it enables high coverage through making use of the best opportunity for immunisation, at the time of birth in a health facility; and it provides the earliest possible protection to infants. This is particularly relevant in places like Papua New Guinea where over half of the deaths from pneumonia in children aged less than 5 years occur in the first six months of life [31]. To achieve early protection we also tried immunising mothers with pneumococcal vaccine in pregnancy [32]. Though this gave encouraging results, it has not yet been followed up with efficacy studies to determine the extent to which maternal immunisation actually protects young infants from disease. A major concern is how to prevent the rapid colonisation of these infants with multiple respiratory pathogens, which is a risk factor for early severe disease — and otitis media — and may also have deleterious immunological effects. A probiotic approach with benign competing organisms would be worth pursuing against both the pneumococcus and haemophilus, and recent clinical [33] and experimental [34] studies with the pneumococcus have shown encouraging results. We would also like to try enhancing mucosal immunity with an infant formulation of the oral haemophilus ‘vaccine’ that we successfully tested in adults.

Boosting of early conjugate vaccine responses by the 23-valent pneumococcal polysaccharide vaccine at 9 months was clearly shown [29]. The positive primary response at this age to serotypes not included in the conjugate vaccine provided further evidence to dispel the myth of the non-immunogenicity of polysaccharide vaccine under the age of 2 years. Similar results were found in Fiji [21], where the functionality of these antibodies was also established [35]. The Fiji study raised the question of hyporesponsiveness following boosting by polysaccharide vaccine [36]. Whether these responses are what one would expect from a well-regulated immune system or lower than expected (hyporesponses) is a matter for debate. Furthermore, it is not clear what the state of ‘hyporesponsiveness’ means, since it is not immunological tolerance in the strict sense. Further detailed investigation of the immune responses, including cellular effects, after pneumococcal antigenic challenge is needed. These aspects are included in the protocol of new studies underway in Papua New Guinea. However, though a high load of polysaccharide antigen does have immunological risks [37] there is no evidence that the dosage of the pneumococcal polysaccharide vaccine used for boosting has been associated with any deleterious clinical effects.

One of the principles of research conducted by the Papua New Guinea Institute of Medical Research has been to ensure access to the best available clinical care for all participants. A further principle is that when an innovative intervention is identified it should be evaluated as soon as possible and, if found to be efficacious, implemented — which, as we have seen, is usually easier said than done. The collective power of the scientists who meet every two years at the International Symposium on Pneumococci and Pneumococcal Diseases (ISPPD) and exchange information on all aspects of pneumococcal science is most impressive. However, there was no mechanism for harnessing this power for the immediate benefit of those who were suffering from pneumonia, the disease most commonly caused by the pneumococcus. We addressed this deficiency at ISPPD5 held in Alice Springs in 2006. A draft Global Action Plan against Pneumonia (GAPP) was presented to and discussed by the Symposium [38]. It became the forerunner of several other Global Action Plans promoted by international agencies. This promotion is active and has expanded to include diarrhoea [39]. However, though good plans make a start, actually getting action is a slow process and can easily lose momentum. The GAPP has remained on the agenda of subsequent ISPPDs and will be discussed again in 2014 in Hyderabad, India. This issue must not be lost sight of and requires continual pushing since pneumonia is ever the forgotten disease or the one that is simply taken for granted. The issue is also surprisingly controversial: when I presented the idea of the GAPP in Alice Springs, the first reaction of a senior colleague was “Why would you want to do that?” Other colleagues were in general supportive of the idea but wanted to push only for vaccines as the means of closing the gap between mortality rates from pneumonia in poor and underprivileged communities and those in affluent ones. All available methods need to be implemented — ready access to good clinical management and appropriate antibiotics when disease occurs and prevention through a healthy environment, good nutrition and vaccines.

One of the most difficult things to achieve in closing this gap is community engagement. Policy-makers and people generally find it difficult to understand that pneumonia is the major killer of children globally and particularly in the hot tropics, where most of these deaths occur. Tropical communities also ignore pneumonia but for the opposite reason: children dying of pneumonia is so common that it is considered a fact of life that one can do nothing about and simply has to accept. To persuade communities and the individuals who live in them that this is not acceptable is a major first step in getting the community engaged in its own health and well-being. Offering accessible clinical care, at a health service facility or in the community, helps, but change will be slow until the community actively pursues ways of improving their own health. Only when the community begins to demand better access to life-saving health care and persists with its demands will deaths from pneumonia significantly decline. Preventive measures such as vaccines and improved household environment and nutrition are part of this care. Enhanced community engagement requires a long-standing dialogue and reciprocal understanding between health services and the community. Strong and committed individuals are needed on both sides. Parents can be taught to recognise when they have a seriously sick child but this recognition has no value if clinical help is not readily accessible. If the staff of a well-equipped clinic have no engagement with the community, there will continue to be frequent deaths from pneumonia in that community. For the future of the community, education and economic opportunity are also vitally important. Nor can the local effects of global environmental change be ignored [40]. To deal with these interfaces we need a new breed of health worker, with similar counterparts in other sectors, and active participation from the community. We have the knowledge and tools already to make a difference to the burden of pneumonia. As well as seeking new knowledge and better tools we need to implement more effectively those that we have. Strengthening community engagement, which extends far beyond pneumonia, marks the final frontier of pneumonia research in the tropics.
